# Epitranscriptomic signatures in blood: emerging biomarkers for diagnosis of diabetes and its complications

**DOI:** 10.3389/fcell.2025.1656769

**Published:** 2025-10-21

**Authors:** Marketa Hlavackova, Daniel Benak, Kristyna Holzerova, Petra Alanova, Jaroslav Hrdlicka, Miloslava Chalupova, Barbora Opletalova, Bohuslav Ostadal, Frantisek Kolar

**Affiliations:** Laboratory of Developmental Cardiology, Institute of Physiology of the Czech Academy of Sciences, Prague, Czechia

**Keywords:** diabetes, biomarkers, epitranscriptomics, RNA modification, m6A, pseudouridine

## Abstract

Type 2 diabetes mellitus (T2DM) is a complex metabolic disorder characterized by chronic hyperglycemia, insulin resistance, and progressive β-cell dysfunction. Traditional biomarkers, such as fasting glucose and glycated hemoglobin (HbA1c), offer diagnostic and prognostic value but have limitations in sensitivity and predictive power for disease progression. Recent advances in molecular biology have identified epitranscriptomic modifications as potential biomarkers for T2DM, offering a novel layer of gene expression regulation through reversible RNA modifications. Dysregulation of these modifications has been implicated in insulin resistance, β-cell failure, and diabetes-related complications. Notably, altered levels of N^6^-methyladenosine (m^6^A) and its regulatory enzymes, including the eraser fat mass and obesity-associated protein (FTO) and the writer methyltransferase-like 3 (METTL3), have been detected in peripheral blood of T2DM patients, suggesting their potential as promising diagnostic markers. Similarly, circulating levels of pseudouridine (Ψ) have been associated with diabetic complications such as retinopathy and nephropathy. This review highlights the emerging role of epitranscriptomic modifications in T2DM pathophysiology and discusses their translational potential as biomarkers for early detection, disease monitoring, and personalized therapeutic strategies.

## 1 Introduction

Diabetes mellitus (DM) is a chronic metabolic disorder characterized by persistent hyperglycemia due to defects in insulin secretion, insulin action, or both. Type 2 diabetes mellitus (T2DM), the most prevalent form, is a growing global health challenge, with its incidence driven by increasing obesity rates, sedentary lifestyles, and aging populations. Given its progressive nature and associated microvascular (including nephropathy, retinopathy, and neuropathy) and macrovascular complications (including cardiovascular disease), early and accurate diagnosis is critical for mitigating long-term morbidity and mortality (Benak et al., 2023a). Current diagnostic and monitoring tools, including fasting glucose, oral glucose tolerance tests, fructosamine, glycated hemoglobin (HbA1c), and glycated albumin have limitations in sensitivity, specificity, and predictive power for disease progression (Dorcely et al., 2017; Ahmed et al., 2025). Consequently, there is an urgent need for novel biomarkers that provide more precise risk stratification and early detection of prediabetes and diabetes.

Recent advancements in molecular biology have expanded biomarker research beyond conventional protein and metabolite markers. The study of post-transcriptional modifications in RNA – referred to as epitranscriptomics or RNA epigenetics – has emerged as a promising frontier in diabetes research (Benak et al., 2023a). Like classical epigenetic modifications, epitranscriptomic modifications also regulate gene expression without altering the nucleotide sequence, offering a dynamic and reversible layer of control over cellular function. Aberrations in RNA modifications have been linked to insulin resistance, β-cell dysfunction, and chronic inflammation – hallmarks of T2DM (Benak et al., 2023a). As such, epitranscriptomic biomarkers hold significant potential as diagnostic and prognostic tools (Santos-Pujol et al., 2024), offering novel insights into disease pathophysiology and paving the way for precision medicine in diabetes management. Moreover, their analysis is no longer limited to advanced LC-MS methods but can often be performed using commercial quantification kits, making them more accessible and economically feasible for routine diagnostic testing.

This short review explores the landscape of epitranscriptomic modifications and their regulators, emphasizing their potential role as biomarkers in T2DM. By integrating this emerging knowledge into clinical practice, we may advance early detection strategies and therapeutic interventions for DM and its complications.

## 2 Epitranscriptomic modifications and their regulators

Epitranscriptomics refers to the study of chemical modifications that occur on RNA molecules, influencing their stability, processing, translation, and degradation (Benak et al., 2024a). Unlike genetic mutations, these modifications are mostly dynamic and reversible, allowing cells to rapidly adapt to physiological and environmental cues. More than 170 distinct RNA modifications have been identified across different RNA species, including messenger RNA (mRNA), transfer RNA (tRNA), ribosomal RNA (rRNA), and non-coding RNAs (ncRNAs) (Cappannini et al., 2024). These modifications play critical roles in regulating cellular metabolism, differentiation, and stress responses – functions that are particularly relevant in the context of DM.

**FIGURE 1:**
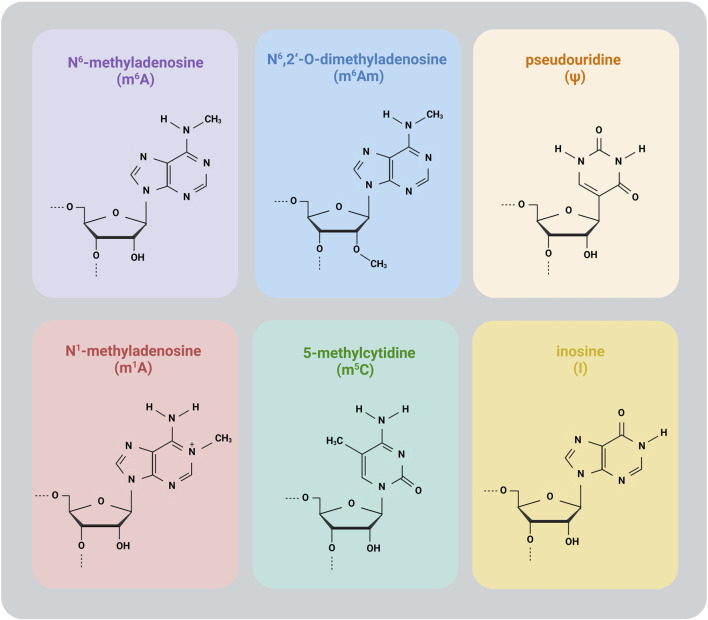
Common RNA modifications.

This review covers the following common modifications: N^6^-methyladenosine (m^6^A), N^6^,2′-O-dimethyladenosine (m^6^Am), N^1^-methyladenosine (m^1^A), 5-methylcytidine (m^5^C), pseudouridine (Ψ) and inosine (I) (Figure 1).

### 2.1 Reversible RNA modifications

Reversible RNA modifications are primarily regulated by three classes of proteins: writers, readers, and erasers. Writers are enzymes that catalyze the addition of specific modifications to RNA, while readers are proteins that recognize and interpret these modifications, mediating downstream effects. Erasers, in turn, remove modifications, creating a dynamic regulatory system (Benak et al., 2024b). These modifications enable cells to respond rapidly and flexibly to cellular signals and environmental changes.

One of the most prevalent RNA modifications in eukaryotic mRNA – and consequently one of the most extensively studied epitranscriptomic modifications – is N^6^-methyladenosine (m^6^A) (Desrosiers et al., 1974; Semenovykh et al., 2022; Benak et al., 2025). This modification plays a crucial role in regulating mRNA stability, splicing, and translation. In addition to mRNA, m^6^A is also present in various other types of RNA (Desrosiers et al., 1974; Dominissini et al., 2013; Meyer et al., 2012; Oerum et al., 2021). The deposition of m^6^A is mediated by a multicomponent methyltransferase complex composed of methyltransferase-like 3 (METTL3), methyltransferase-like 14 (METTL14), and Wilms’ tumor 1-associating protein (WTAP) (Wan et al., 2016; Wang et al., 2016). Recognition of m^6^A is facilitated by a variety of m^6^A-binding proteins, including YTH domain-containing family proteins (YTHDF1-3) (Zaccara and Jaffrey, 2020; Lasman et al., 2020; Wang et al., 2014; Wang et al., 2015; Shi et al., 2017), YTH domain-containing proteins (YTHDC1-2) (Xiao et al., 2016; Hsu et al., 2017; Ping et al., 2014), insulin-like growth factor 2 mRNA-binding proteins (IGF2BP1-3) (Huang et al., 2018), and heterogeneous nuclear ribonucleoproteins (HNRNPA2B1, HNRNPC, HNRNPD, HNRNPG) (Alarcón et al., 2015; Liu et al., 2015; Song et al., 2019; Liu et al., 2017). The removal of m^6^A is carried out by demethylases such as fat mass and obesity-associated protein (FTO) (Jia et al., 2011; Benak et al., 2024c) and AlkB homolog 5 (ALKBH5) (Zheng et al., 2013; Wang et al., 2023a). Notably, dysregulation of m^6^A and its regulators has been observed in various diabetic tissues. This topic has been reviewed in detail (Benak et al., 2023a).

N^6^,2′-O-dimethyladenosine (m^6^Am) differs from m^6^A by the presence of an additional 2′-O-methyl group. In mRNA, m^6^Am is predominantly found at the mRNA cap, positioned at the transcription start site adjacent to the 7-methylguanosine (m^7^G) cap structure (Wei et al., 1975; Bokar and Grosjean, 2005). In small nuclear RNA (snRNA), m^6^Am also occurs at internal sites, where it contributes to pre-mRNA splicing (Mauer et al., 2019). The cap-associated m^6^Am is introduced by phosphorylated CTD-interacting factor 1 (PCIF1) (Akichika et al., 2019; Sun et al., 2019), whereas methyltransferase-like 4 (METTL4) catalyzes its incorporation at internal snRNA sites (Goh et al., 2020; Chen et al., 2020). Currently, no m^6^Am-specific readers have been identified, and only a single eraser is known to remove its N^6^-methylation – the well-characterized m^6^A demethylase FTO. Studies suggest that FTO predominantly demethylates m^6^Am in the cytosol, whereas in the nucleus, its primary target is m^6^A (Wei et al., 2018; Benak et al., 2023b). The relationship between m^6^Am and diabetes remains unclear. However, since many detection methods do not differentiate between m^6^A and m^6^Am (Benak et al., 2023b), and the well-studied FTO enzyme acts on both modifications (Benak et al., 2024c), m^6^Am is included in this review.

N^1^-methyladenosine (m^1^A) is predominantly found in tRNA and rRNA, with a lower abundance in mRNA (Dunn, 1961; Helm et al., 1999; Sharma et al., 2013; Dominissini et al., 2016). Functionally, it influences the structure and stability of tRNA and rRNA, while in mRNA, it plays a role in regulating translation (Dominissini et al., 2016; Oerum et al., 2017; Shima and Igarashi, 2020; Safra et al., 2017; Zhao et al., 2017). Its methylation is catalyzed by tRNA methyltransferase 6 (TRMT6), TRMT61A, TRMT61B, TRMT10C, and ribosomal RNA-processing protein 8 (RRP8, also known as NML) (Safra et al., 2017; Li et al., 2017; Chujo and Suzuki, 2012; Bar-Yaacov et al., 2016; Waku et al., 2016). The demethylation of m^1^A is carried out by the erasers ALKBH1 and ALKBH3 (Dominissini et al., 2016; Liu et al., 2016; Li et al., 2016a; Chen et al., 2019a). Additionally, FTO, primarily known as an m^6^A and m^6^Am eraser also acts as an m^1^A demethylase in tRNA (Wei et al., 2018). The link between m^1^A and diabetes remains unclear. However, ALKBH1, an m^1^A demethylase, was found to be downregulated in pancreatic islet samples from T2DM patients (Wu et al., 2023).

5-methylcytidine (m^5^C) is a widely distributed RNA modification found across multiple RNA types. It plays a crucial role in regulating RNA export, ribosome biogenesis, translation, and RNA stability (Bohnsack et al., 2019; Squires and Preiss, 2010; Chen et al., 2021). In humans, m^5^C is deposited by the NOL1/NOP2/SUN domain (NSUN) family proteins (NSUN1-7) as well as DNA methyltransferase homolog DNMT2 (also known as TRDMT1) (Bohnsack et al., 2019; Wang et al., 2023b). Among the m^5^C-binding proteins, Aly/REF export factor (ALYREF) facilitates nuclear-to-cytoplasmic RNA transport (Yang et al., 2017), whereas Y-box-binding protein 1 (YBX1) stabilizes its target mRNAs by interacting with ELAVL1 (Chen et al., 2019b). The removal of m^5^C is mediated by ten-eleven translocation (TET) proteins (TET1-3) and ALKBH1. The TET enzymes catalyze the oxidation of m^5^C to 5-hydroxymethylcytidine (hm^5^C), while ALKBH1 specifically oxidizes m^5^C in mitochondrial tRNA, generating 5-formylcytidine (f^5^C) (Haag et al., 2016; Fu et al., 2014). Notably, 5-methylcytosine also occurs in DNA, where it is often referred to as 5mC. Although the regulatory mechanisms of this modification differ between DNA and RNA, they share certain modifying enzymes, particularly TET proteins, which have been extensively studied in DNA demethylation (Williams et al., 2011). In the context of diabetes, a recent study found that m^5^C-related genes were significantly differentially expressed in T2DM and showed strong correlations with the majority of T2DM-associated differentially expressed genes in skeletal muscle samples (Song et al., 2022). The m^5^C reader NSUN2 has been linked to diabetic retinopathy (Wang et al., 2024) and nephropathy (Wang et al., 2025). Additionally, increased expression of *Nsun4*, *Nsun6*, and *Dnmt2* has been observed in diabetic retinopathy (Wang et al., 2023c). Berberine, a compound known for its protective effects against diabetic nephropathy, has been reported to suppress DNMT2 expression in diabetic nephropathy mouse models (Cai et al., 2024). The m^5^C eraser *TET1* was downregulated in human pancreatic islets from T2DM patients (Bacos et al., 2023) as well as in renal tissues of diabetic nephropathy mouse models (Tan et al., 2021). Another recent study showed that proteins TET1-3 play a critical role in *de novo* blood vessel formation, aiding the rescue of diabetic ischemic skin (Mohanty et al., 2024). Finally, as previously mentioned, ALKBH1 – a demethylase of both m^1^A and m^5^C – was found to be downregulated in pancreatic islet samples from T2DM patients (Li et al., 2016a).

### 2.2 Irreversible RNA modifications

Unlike reversible RNA modifications, irreversible modifications lack erasers that could dynamically regulate their presence in RNA, thereby limiting their regulation to mRNA turnover.

Pseudouridine (Ψ), a C5-glycoside isomer of uridine (U), was the first RNA modification ever discovered and remains the most abundant, detected across nearly all types of RNA (Cohn, 1951; Xue et al., 2022; Sun et al., 2023). Functionally, Ψ plays a key role in stabilizing RNA structures while simultaneously reducing RNA-binding protein interactions. In mRNA, its most studied role is enhancing stop codon read-through (Sun et al., 2023; Borchardt et al., 2020). The enzymatic conversion of U to Ψ is catalyzed by the pseudouridine synthase (PUS) family, a diverse group of enzymes responsible for this modification (Rintala-Dempsey and Kothe, 2017). To date, 13 PUS enzymes have been identified in eukaryotes (Sun et al., 2023). In humans, this family includes PUS1, PUS3, PUS7, PUS10, PUSL1, PUSL7, TRUB1-2 (TruB pseudouridine synthase 1-2), RPUSD1-4 (RNA pseudouridine synthase D1-4), and DKC1 (dyskerin pseudouridine synthase 1) (Li et al., 2016b). Currently, the only known Ψ-binding protein is the yeast RNA helicase Prp5, which interacts with snRNA (Wu et al., 2016; Levi and Arava, 2021). Diabetic complications, such as diabetic retinopathy and diabetic nephropathy, have been associated with changes in circulating Ψ levels (Sun et al., 2021; Jiang et al., 2024; Mathew et al., 2024; Niewczas et al., 2017); however, the link between Ψ and its regulators in diabetes remains unknown.

Inosine is a product of A-to-I editing, a conserved mechanism that contributes to transcriptome diversity as part of the broader RNA editing process, which also encompasses cytosine-to-uridine conversion and nucleotide insertions and deletions (Brennicke et al., 1999; Gott and Emeson, 2000). This modification occurs when the C^6^-position of adenosine loses a hydrogen-donating amino group, resulting in inosine, which structurally resembles guanosine and can influence various downstream processes. Post-transcriptionally, A-to-I editing can alter codons, create or eliminate splice sites, modify microRNA (miRNA) interactions, and influence RNA base pairing with itself or other RNAs, as well as its binding to RNA-associated proteins. In coding regions, this process can lead to amino acid substitutions, potentially affecting protein function (Nishikura, 2016). Deamination of adenosine to inosine is performed by enzymes belonging to the adenosine deaminase acting on RNA (ADAR) family, which is represented by three ADAR orthologs (ADAR1-3) in mammals. ADAR1 and ADAR2 are widely expressed, while ADAR3 was detected only in the brain (Ganem and Lamm, 2017; Dominis et al., 2011). Both mouse and human β-cells require intact ADAR1 function, as its disruption leads to the accumulation of endogenous double-stranded RNA (dsRNA), activation of an interferon response, islet inflammation, and β-cell failure. These changes closely mimic key aspects of early-stage T1DM (Kneb et al., 2024). Interestingly, inosine supplementation has been reported to protect against T1DM by exerting anti-inflammatory effects and modulating immune responses (Mabley et al., 2003). However, these effects appear to be independent of inosine’s role in RNA editing and are instead linked to its function as a purine metabolite.

## 3 Epitranscriptomic biomarkers in diabetic patients

Epitranscriptomic modifications have emerged as potential biomarkers for T2DM. Changes in their levels and the expression of its regulatory enzymes in peripheral blood may reflect disease progression and metabolic dysregulation, making them promising candidates for novel diagnostic tools.

**FIGURE 2:**
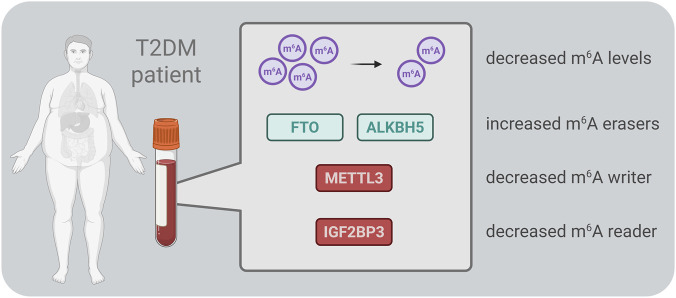
Schematic overview of the main m^6^A-related enzymes reported in blood samples from patients with type 2 diabetes mellitus (T2DM). ALKBH5 – AlkB homolog 5, FTO – fat mass and obesity-associated protein, IGF2BP3 – insulin-like growth factor 2 mRNA-binding protein 3, METTL3 – methyltransferase-like 3.

Decreased m^6^A methylation levels have been reported in RNA isolated from the peripheral blood of T2DM patients and diabetic rats (Shen et al., 2015; Onalan et al., 2022). Consistent with this, *FTO* gene expression – but not *ALKBH5* – was found to be significantly upregulated in peripheral blood from T2DM patients (Shen et al., 2015). However, a separate study by Onalan et al. (Onalan et al., 2022) observed increased expression of both demethylases in venous blood samples from T2DM patients. Further supporting the role of FTO, another study confirmed its elevated expression at both gene and protein levels, highlighting a correlation between high FTO levels and T2DM severity (Masoud Abd El Gayed et al., 2021). Similarly, *FTO* gene expression was upregulated in white blood cells of T2DM patients compared to healthy individuals, with its expression level positively correlated with fasting glucose concentration (Yang et al., 2019). Apart from m^6^A erasers, *METTL3*, a key m^6^A methyltransferase, was found to be downregulated in serum samples from T2DM patients (Zha et al., 2020). Additionally, low serum levels of IGF2BP3, an m^6^A reader, were associated with a progressively higher risk of developing T2DM (Wu et al., 2023). Collectively, these findings suggest that m^6^A modifications and their regulatory proteins in peripheral blood could serve as novel epitranscriptomic biomarkers for T2DM (Figure 2). Their potential use in early diagnosis, disease monitoring, and risk assessment warrants further investigation.

Additionally, Ψ has recently been identified as a circulating biomarker related to diabetes complications. Elevated Ψ levels have been associated with the occurrence of diabetic retinopathy (Sun et al., 2021) and have been identified as an early biomarker of diabetic kidney disease in Chinese patients with T2DM (Jiang et al., 2024). Moreover, Ψ levels have been linked to renal function decline and the progression to end-stage renal disease in patients with type 1 diabetes mellitus (T1DM) (Niewczas et al., 2017).

Other RNA modifications and their regulatory enzymes may also play a role in diabetes and its complications, but they remain largely unexplored as potential biomarkers. For example, m^1^A, m^5^C, and inosine are among the modifications that have been linked to diabetes-related processes but have yet to be studied in the context of their potential as diagnostic or prognostic biomarkers.

Importantly, circulating alterations in RNA modifications seem unlikely to exert direct pathogenic effects themselves but rather serve as biomarkers that mirror dysregulated processes in tissues such as pancreatic islets, liver, or kidney. Establishing these tissue–blood relationships will be essential for clarifying underlying mechanisms and for translating biomarker discovery into therapeutic strategies. To this end, integrating blood- and tissue-level epitranscriptomic analyses could refine our understanding of disease pathogenesis, uncover organ-specific vulnerabilities, and guide the development of more precise interventions. Such a dual approach carries translational potential by directly linking biomarker discovery to drug development. Future research should also focus on expanding the scope of epitranscriptomic biomarkers beyond m^6^A to include m^1^A, m^5^C, inosine, and Ψ, as their regulatory mechanisms and clinical significance in DM remain largely unexplored. Elucidating how these modifications influence β-cell function, insulin resistance, and inflammation may open new avenues for early detection, disease monitoring, and therapeutic intervention in DM and its complications.

## 4 Conclusion

Epitranscriptomic modifications represent a promising frontier in diabetes biomarker research, providing dynamic and often reversible regulation of RNA metabolism. The emerging evidence linking RNA modifications to insulin resistance and β-cell dysfunction underscores their potential as novel diagnostic and prognostic tools. While m^6^A modifications have been most extensively studied in diabetes, the broader landscape of RNA modifications remains largely unexplored. Future research should focus on validating these biomarkers in large patient cohorts, understanding their mechanistic roles in diabetes pathophysiology, and developing clinically feasible detection methods. Integration of epitranscriptomic signatures into precision medicine approaches may ultimately enhance early diagnosis, risk stratification, and personalized therapeutic interventions in T2DM.
